# Different Contexts, Similar Challenges. SUSTAIN’s Experiences with Improving Integrated Care in Europe

**DOI:** 10.5334/ijic.5492

**Published:** 2020-06-26

**Authors:** Simone R. de Bruin, Jenny Billings, Annerieke Stoop, Manon Lette, Eliva A. Ambugo, Erica Gadsby, Christina Häusler, Konrad Obermann, Gerli-Paat Ahi, Jillian Reynolds, Georg Ruppe, Nhu Tram, Gerald Wistow, Nick Zonneveld, Giel Nijpels, Caroline Baan, On behalf of the SUSTAIN consortium

**Affiliations:** 1 National Institute for Public Health and the Environment, Bilthoven, NL; 2 Integrated Care Research Unit Centre for Health Service Studies, University of Kent, Canterbury, UK; 3 Amsterdam Public Health research institute, Department of General Practice and Elderly Care Medicine, Amsterdam UMC – VU University Amsterdam, Amsterdam, NL; 4 Scientific Centre for Transformation in Care and Welfare (Tranzo), University of Tilburg, Tilburg, NL; 5 Department of Health Management and Health Economics, Institute of Health and Society, University of Oslo, Oslo, NO; 6 Centre for Health Services Studies, University of Kent, Canterbury, UK; 7 Austrian Interdisciplinary Platform on Ageing/OEPIA, Vienna, AT; 8 Mannheim Institute of Public Health (MIPH), Heidelberg University, DE; 9 Praxis Centre for Policy Studies Foundation, Tallinn, EE; 10 Agency for Health Quality and Assessment of Catalonia (AQuAS), Barcelona, ES; 11 AGE Platform Europe, Brussels, BE; 12 Personal Social Services Research Unit, Department of Social Policy, London School of Economics and Political Science, London, UK; 13 National Centre of Excellence in Long Term Care, Utrecht, NL; 14 TIAS School for Business and Society, University of Tilburg, Tilburg, NL

**Keywords:** European project, integrated care, older people, participatory research

In 2019, we finalized a four-year EU-funded research project on integrated care for older people called ‘SUSTAIN’ (see **Textbox 1** below). We initiated the project to help address the challenges faced by those practicing integrated care, including how to successfully collaborate with organisations and professionals from different sectors; how to incorporate integrated working in one’s service delivery; and how to provide integrated care tailored to the needs of one’s older service users [1,2,3,4,5]. The SUSTAIN project was designed to support the development of integrated care for older people living at home with health and social care needs. Its objectives were twofold: 1. To support and monitor improvements to established integrated care initiatives and 2. To contribute to the adoption and application of such improvements to other health and social care systems and regions in Europe. The project adopted a participatory implementation approach, meaning that stakeholders from thirteen established integrated care initiatives across Europe collaborated with SUSTAIN researchers to develop and implement a wide variety of activities to improve integrated care delivery within a number of domains: person-centredness, prevention-orientation, safety, efficiency, and coordination. Throughout the project, SUSTAIN researchers evaluated progress and outcomes of these improvement processes [6].

**Box 1: The SUSTAIN-project.**

SUSTAIN stands for ‘**Sus**tainable **Ta**ilored **In**tegrated Care for Older People in Europe’ (www.sustain-eu.org). The SUSTAIN-project was carried out between 2015 and 2019 by thirteen partners from eight European countries: Austria, Belgium, Estonia, Germany, Norway, Spain (Catalonia), the Netherlands, and the United Kingdom. With the exception of partners in Belgium, all SUSTAIN researchers selected two integrated care initiatives in their countries for participation in the SUSTAIN-project. The different integrated care initiatives were committed to improving their current practices by working towards more person-centered, prevention-oriented, safe, efficient, and coordinated care. The integrated care initiatives served different target groups and provided various types of care services, including proactive primary and social care for frail older people, care for older people being discharged from hospital, care for people with dementia, and home nursing and rehabilitative care.

The SUSTAIN researchers supported local steering groups, consisting of stakeholders from different local organisations (e.g. GP practice, hospital, home care organisation, social care organisation, municipality, advocacy organisation for older people) at the different initiatives to design and implement plans to improve their current ways of working. Plans consisted of sets of activities to enhance various aspects of integrated care and reflected the priorities of local stakeholders. The SUSTAIN researchers evaluated the progress and outcomes of these improvement processes.

SUSTAIN’s main deliverable was a roadmap to support policy-makers, decision-makers and health and social care professionals, with several tools and guides in implementing and improving integrated care (https://www.sustain-eu.org/wp-content/uploads/sites/4/2019/03/SUSTAIN-Roadmap.pdf).

In addition to a range of scientific papers, the project further produced a series of reports on the improvement processes in the different countries (https://www.sustain-eu.org/products/sustain-country-reports/).

*The project was funded under Horizon 2020 – the Framework Programme for Research and Innovation (2014–2020) from the European Commission (EC) under grant agreement No. 634144. The SUSTAIN project was initiated in April 2015 and ended in March 2019. The content of this article reflects only the SUSTAIN consortium members’ views. The European Union is not liable for any use that may be made of the information contained herein*.

The project has generated lots of insights into and experiences with integrated care implementation and evaluation [7,8]. This special IJIC issue on SUSTAIN features six papers covering a variety of aspects of integrated care, including the implementation of activities to improve integrated care [9,10,11], the exploration of experiences of different stakeholders with integrated care, i.e. service users, informal carers, professionals, and managers [9,11,12,13,14], and the evaluation of integrated care by means of a Patient Reported Outcome Measure (PREM) [14]. The SUSTAIN project generated several overall lessons from its experiences of both developing and evaluating integrated care improvements [7]. This special issue highlights four of these lessons. The lessons are briefly introduced below and further illustrated in the individual papers.

A first lesson that we learned is, that despite differences in their characteristics (e.g. settings they operated within; care and support services that they provided; characteristics of their target populations) and the contexts within which they operated (e.g. national legislation and funding; region’s readiness for integrated care), integrated care initiatives across Europe experienced quite similar challenges with improving their services. These challenges were similar to those that have been found in other studies on transformations of health and social care systems [15,16,17]. Furthermore, the challenges are complex and concern different levels of health and social care systems (e.g. level of individual service user, level of teams of health and social care professionals, level of different health and social care organisations, political and economical environment). Examples of these challenges include [7,8]:

- Lack of appropriate payment models for integrated care;
- Lack of successful governance arrangements, including aspects such as accountability and leadership;
- Lack of commitment of different health and social care providers or agencies involved in the integrated care initiative, and lack of a shared vision;
- Lack of clearly defined and allocated roles and responsibilities of the health and social care professionals involved;
- Lack of trust and understanding of health and social care providers of one another’s norms, values, roles and expertise;
- Insufficient information sharing within and between organisations, providers, and service users;
- Difficulties in tailoring services to the needs and wishes of the older person.

These challenges are often interrelated, which means that tackling these challenges involves efforts on multiple levels of health and social care systems and in multiple areas within these levels. This special issue features three papers that present activities that were developed and implemented to tackle some of these challenges. The papers by Stoop et al. [11] and Lette et al. [9] review all integrated care initiatives that participated in SUSTAIN, reflecting activities that were undertaken by the stakeholders of the initiatives to improve person-centredness and safety respectively. The paper by MacInnes et al. [10] focusses on the improvement process of one specific integrated care initiative in the United Kingdom.

The finding that there is a gap between managers’ and professionals’ views and those of users and informal carers on essential aspects of integrated care is a second lesson the SUSTAIN team learned. In the SUSTAIN project, stakeholders from the different integrated care initiatives undertook a wide range of improvement activities to improve their services, as was already mentioned under lesson 1. Examples include building a multidisciplinary team, developing tools for needs assessment and care planning, doing home safety assessments, organizing training on interprofessional communication and collaborating, and empowering users and their informal carers to play a role in healthcare decisions. The studies of Stoop et al. [11] and Lette et al. [9], which are featured in this special issue, reflect on these improvement activities and address experiences with these activities from multiple perspectives, i.e. the perspectives of managers and health and social care providers as well as older people and their informal carers. The study of Ambugo et al. [12] looked into the experiences of informal carers with integrated care more in-depth. From these studies, we learned that the experiences of managers and professionals were generally more favorable than those of users and informal carers. For instance, managers and professionals reported experiences of improved person-centered working (e.g. working with a care plan) and enhanced safety at home (e.g. provision of safety information and advice to support independent living, given in the user’s own home). However, older people and informal carers often did not find these activities relevant, had not noticed them, or did not perceive them as an improvement. These conclusions imply that there is a mismatch between what managers and professionals think are important aspects of integrated care and how users and informal carers understand, evaluate or perhaps prioritise them.

A third lesson that we learned is that the process of improving integrated care should be conceptualized and experienced as a learning cycle. The integration of health and social care is a complex process that is (as also outlined under lesson 1) dependent on a plethora of factors. [17,18,19,20,21]. In SUSTAIN, we therefore used participatory implementation research to support local stakeholders to improve their existing ways of working. The paper by MacInnes et al. [10] focusses on the improvement process of one specific integrated care initiative in the United Kingdom. SUSTAIN showed us that collaborative approaches, that use an iterative cycle of research, action, and reflection, are necessary to implement and improve integrated care successfully. One should, however, realize that these processes of collaboration, co-creation, learning, and reflection take time. Taking small steps and building on existing experiences and practice will help leverage commitment to the change process and thereby support its feasibility and sustainability [7,22].

The finding that integrated care evaluation in a cross-European project remains challenging is a fourth lesson learned [7]. As in previous studies [16,23], we learned that this is challenging for several reasons. For instance, there are ongoing debates about how to evaluate integrated care (i.e. moving away from traditional designs and indicators). Furthermore, there is a lack of shared understanding of methodological approaches across researchers due to different disciplinary backgrounds, data collection skills and availability of data [7]. We realized that current methodologies are often insufficient in dealing with the complexity of initiatives such as integrated care, and their interaction with national, regional and local contextual factors [16,24]. Related to this is a lack of appropriate indicators to measure the impact of integrated working on the level of the service user. Several studies have proposed innovative and meaningful indicators to measure service users’ experiences and impact of integrated care delivery [25,26]. In SUSTAIN, we used the innovative Person Centred Coordinated Care Experience Questionnaire (P3CEQ) instrument which has explicitly been developed to measure the experiences of people with long-term conditions (e.g. older people with complex needs) with integrated care delivery [27]. Not withstanding its merits, we also encountered some challenges using this instrument that illustrate the complexity of capturing the voice of vulnerable target groups concerning complex constructs which are described in the paper by Reynolds et al. [14].

## Conclusion

In the EU, initiatives in the area of integrated care are widespread, though the characteristics and contexts of these initiatives vary significantly. Policy-makers, service providers, and researchers keep looking for better ways to set up new services and improve existing ones – with the ultimate goals of improving people’s experiences of care delivery, enhancing care outcomes, limiting health and social care costs, and improving the working lives of health and social care professionals. By evaluating the structures, processes and outcomes of improvement initiatives in different countries, SUSTAIN obtained insights into what has and has not worked, when implementing improvements to integrated care initiatives. We learned that it is often about getting the right people with the right energy to devote time to finding ways of critically assessing their ways of working, seeing things through other perspectives (i.e. other care providers’, the older persons’, the carers’) and jointly identifying and being brave enough to make use of windows of opportunity to make change happen despite the well-known barriers. By sharing our learning in this special issue on the SUSTAIN project, we hope to provide starting-points for policy-makers, service providers, and the research community to better shape future integrated care policy and delivery.
